# Translational simulation revisited: an evolving conceptual model for the contribution of simulation to healthcare quality and safety

**DOI:** 10.1186/s41077-024-00291-6

**Published:** 2024-05-08

**Authors:** Victoria Brazil, Gabriel Reedy

**Affiliations:** 1 Faculty of Health Sciences and Medicine, Bond University, Gold Coast, QLD Australia; 2 Faculty of Life Sciences and Medicine, King’s College London, Waterloo Bridge Wing G7, London, UK

**Keywords:** Translational Simulation, Healthcare simulation, Quality improvement, Patient safety, In situ simulation

## Abstract

The simulation community has effectively responded to calls for a more direct contribution by simulation to healthcare quality and safety, and clearer alignment with health service priorities, but the conceptual framing of this contribution has been vague. The term ‘translational simulation’ was proposed in 2017 as a “*functional term for how simulation may be connected directly with health service priorities and patient outcomes, through interventional and diagnostic functions*” (Brazil V. Adv Simul. 2:20, 2017). Six years later, this conceptual framing is clearer. Translational simulation has been applied in diverse contexts, affording insights into its strengths and limitations. Three core concepts are identifiable in recently published translational simulation studies: a clear identification of simulation purpose, an articulation of the simulation process, and an engagement with the conceptual foundations of translational simulation practice. In this article, we reflect on current translational simulation practice and scholarship, especially with respect to these three core concepts, and offer a further elaborated conceptual model based on its use to date.

## Background

The healthcare simulation community was quick to step into a role in healthcare quality and patient safety in the late 1990s. Simulation offers a safe place to practice procedural skills, decision-making and teamwork without placing patients at risk. Education can be ‘on demand,’ scheduled to suit learners and teachers, without relying on opportunistic clinical encounters. Simulation can afford high-volume practice with feedback. Exemplar simulation programs have demonstrated measurable impacts on safety and quality outcomes [1–3]. Simulation designed and delivered within this educational paradigm continues to have an important role in supporting healthcare quality and safety.

However, reliance on educational paradigms may fail to realise the full potential of simulation to contribute to quality and safety in healthcare. Healthcare operates as a complex adaptive system, with rich interdependencies between providers, structural elements and social systems [4]. Performance—healthcare that is safe, effective, timely, patient-centred, efficient and equitable [5]—is reliant on more than the knowledge and skills of individuals or teams. For example, training nurses on the dangers of rapid intravenous potassium administration may not be enough to reduce adverse events until concentrated potassium ampoules are removed from wards and replaced with 500 ml bags of diluted potassium [6]. Knowing which blood products are required for a trauma patient will not be enough if there are ineffective systems for ordering and delivering those blood products to the trauma bay [7, 8]. Teaching healthcare practitioners to ‘speak up for safety’ will be ineffective if toxic hierarchies and cultural norms go unaddressed in healthcare environments [9]. Hence, an educational approach to improving patient safety is necessary, but insufficient, to fulfil the potential for simulation to contribute to improved healthcare outcomes [10].

In the article "Translational Simulation—not where but why" [11], terminology was suggested to reflect a revised conceptual framing for the contribution of simulation to healthcare quality and safety. Drawing on Berwick’s ‘Plan Do Study Act’ framework for quality improvement activities [12], this conceptual model illustrated an expanded range of opportunities that simulation practitioners should embrace for the purpose of improving patient care and health systems. Many of these opportunities had not been considered within the remit of healthcare simulation, which had previously focused largely on the education and training of health professionals. A structured approach to how health service outcomes might be achieved through simulation was described: simulation can be used to *explore* performance, and to *test and embed* improvements in systems and processes. Through these diagnostic and interventional functions, the scope of simulation in improving health service performance could be widened to include outcomes such as timeliness of care, cost-effectiveness, patient experience, efficiency of care, effectiveness of clinical pathways, team culture and adequacy of the physical environment [11].

The term ‘translational simulation’ drew upon language from the biological sciences research context, where ‘translational’ refers to basic science research evidence being applied to real-world practice. Framing the “bench-to-bedside enterprise of harnessing knowledge from basic sciences to produce new drugs, devices, and treatment options for patients” [13] as a translational activity has focused biomedical researchers on their ultimate purpose—the health of patients and populations.

“All models are wrong, but some are useful” [14]. With this aphorism in mind, we might ask: Why do we (or *why does the field*) need a conceptual framing for how simulation contributes to healthcare quality and safety, and its ongoing evolution? We suggest that a foundational framework would provide numerous benefits for health service leaders, healthcare simulation practitioners, and scholars. For health services, explicitly framing how healthcare simulation contributes to quality and safety can guide health service resource allocation and optimise the use of simulation capacity. Clearer framing may help health service leaders revise the view of simulation-based education as a financial burden on healthcare institutions, and instead recognise the value of simulation to address organisation and system objectives [15]. For simulation practitioners, a conceptual framing may provide a common language, and guide programs seeking to articulate their mission, vision and scope [15]. Emphasizing the ‘why’ of translational simulation activities—healthcare improvement—may encourage reflection on the methods and tools used in simulation design and delivery [16]. For scholars, a clear conceptual model supports voices arguing for explicit integration of theory as a *“conceptual and framing device”* within simulation research [17].

We propose that the conceptual framing for translational simulation has evolved since 2017 [11]. In the subsequent sections of this article, we will explore the application of translational simulation in practice, while critiquing the strengths and limitations of this emerging conceptual framing. We will consider alternative terminology and framing presented in the literature: in situ simulation (ISS), non-pedagogical simulation, simulation-based clinical systems testing, and systems integration simulation. We then present a revised conceptual model, representing our synthesis and critical review of scholarship in this area. The model is comprised of three elements—purpose, process and conceptual foundations—that we have identified in reflecting on translational simulation scholarship and practice. Finally, we reflect on directions for future practice and research.

## Exploring translational simulation in practice

An analysis of the literature citing Brazil’s translational simulation article [11] demonstrates diverse applications of the concept. These include translational simulation to prepare health services for the COVID-19 pandemic [18–30], for hospital relocation or physical space testing [31], for clinical pathway or process testing [32–39], and for shaping culture and teamwork in healthcare settings [35, 40–46]. Other citing literature has included conceptual discussions and review articles [47–51], as well as efforts to develop methods and tools for ‘translational simulation in action’ [16, 32, 52]. We now consider and critique these examples and provide a summary in Table 1.

**Table 1:** Exploring translational simulation in practice-examples

Application	Examples
Responding to COVID-19	Modified guidelines and processes for cardiac arrest, airway management, maternity care, patient triage when healthcare resources were overwhelmed. Testing novel devices for ‘COVID safe’ procedures.
Testing clinical processes	Optimizing airway emergency cart design. Reducing time to intervention for stroke and myocardial infarction patients. Reducing time to transfer to the operating theatre. Improved multidisciplinary response to paediatric anaphylaxis.
Designing physical infrastructure	New building design Human-centred device/equipment design
Building teams, shaping culture and relationships	Major trauma care, operating theatre teams, maternity emergencies Identifying ‘latent social threats’. Building rituals for team-based performance reflection.
Supporting healthcare improvement	Exploring and shaping the context of care Research test bed

### Responding to COVID-19

Responding to COVID-19 offered an exemplar of how translational simulation could contribute to healthcare process re-design, especially under conditions that were fast-paced and high stakes [18, 22, 27, 29]. A clear lesson, from experience around the world, was that health services should have a strategy and capacity to use translational simulation to enable rapid responsiveness to a crisis [29]. Dube et al. described a large-scale effort to use simulation to prepare for COVID-19 across the province of Alberta, Canada, illustrating the role of simulation in *organizational* learning [18]. Numerous specific examples of how simulation was used to develop and adapt clinical care for COVID-19 were published, including the development of modified guidelines for cardiac arrest [53], airway management [54], maternity care [25], and patient triage when healthcare resources may be overwhelmed [55]. 

The global COVID-19 pandemic offered a unique context for the application of translational simulation, with lessons that extended beyond that context. For many health services and simulation practitioners it was the first time that simulation has been so closely applied to health service priorities and used for rapid adaptation of systems and processes. While educationally-focused simulation programs were closing their doors due to COVID restrictions, translational simulation activities ramped up, with unprecedented volumes of activity [18, 22, 25, 29, 30]. 

When viewed from the perspective of our conceptual model for translational simulation, our reflections on these published examples of experience during the COVID-19 pandemic are twofold. First, the disciplined focus on *purpose* remains well placed in the model—aligning with present and emerging health service priorities. Second, a gap in guidance on the *process* can be identified. Many simulation programs struggled to adapt their simulation design, delivery and debriefing to the novel purpose of system and process testing. Drawing on methods from educationally focused simulations did not always provide the guidance needed to achieve optimal process re-design [22]. By contrast, successful initiatives were reported in programs that had established methods and tools drawn from quality improvement, systems engineering and process redesign [18, 19, 22, 30]. The *conceptual foundations* of these practice fields were rarely explicitly integrated or articulated in the haste of simulation practice and publication during the COVID-19 pandemic.

### Testing clinical processes

The efficiency, safety and effectiveness of patient care journeys have provided fertile ground for healthcare improvement using translational simulation. Examples include: introducing ward-level high-flow oxygen care for infants with bronchiolitis [39], optimization of paediatric airway emergency carts to improve response times in emergencies [56], improved time to intervention in acute stroke [57], improved multidisciplinary response to anaphylaxis in the paediatric emergency department [58], improving rapid transfer to the operating theatre for critically unwell trauma patients at a tertiary referral hospital [59] and reducing the ‘door to needle’ time for patients suffering a myocardial infarction who required safe and fast transfer from the emergency department to cardiac catheter suite [60].

Our reflection on these published examples highlights that most are context-specific case studies [60–64], with simulation methods drawn from educational contexts or reliant on local resources and capacity. This further highlights a limitation of translational simulation: while it represents a broad conceptual reframing of how simulation can contribute to healthcare improvement, it lacks a clear *process* by which these aims are achieved.

### Designing physical infrastructure

Testing the adequacy of physical infrastructure using simulation is not a new concept, but has been surprisingly underutilised in the design and building of healthcare facilities [47, 65]. Guidance has been offered on optimal simulation design for practitioners seeking to test physical infrastructure. For example, in reporting experience in testing new building designs, Barlow et al. offer a documentation framework for healthcare simulation quality improvement activities [66]. Their framework draws upon established methods for quality improvement, including the Failure Modes Effects Analysis (FMEA) approach [67] to collecting and analysing data from translational simulation activities. Seeking to develop a standardised approach to systems testing, Colman et. al. presented a ‘Simulation-based clinical systems testing’ (SbCST) framework, including documentation and evaluation tools [68]. Drawing upon a different conceptual framework, Petrosoniak et al. describe a ‘design thinking-informed’ simulation framework to test, evaluate, and modify new clinical infrastructure [69]. This included the key features (and language) of design thinking [70]: end-user engagement, rapid prototyping and testing, and an experimentation mindset. Kaba et al. offer lessons learned from using process-orientated simulations to test the opening of a new 300-bed healthcare facility [71]. Although offering diverse methodological approaches, these conversations shared a common stance—that the incorporation of simulation and human factors into hospital design is essential [65]. 

Our reflection on these applications of translational simulation principles identifies an additional gap in the original framing of translational simulation. Broader conceptual foundations—from design theory, human factors and ergonomics, change management, and systems engineering—are necessary to develop the methods and tools for this use of simulation to be effective, and should be explicitly articulated in a revised conceptual framing.

### Building teams, shaping culture and relationships

Improvements in healthcare teamwork have been demonstrated in many simulation activities. Less frequent has been a deliberate, systematic focus on teamwork, relationships and culture within intact teams in healthcare institutions. Translational simulation embraces these outcomes as central to improving health service performance. Published examples include shaping relationships and culture in major trauma care [40, 42], maternity emergencies [44], neonatology trainees ‘boot camp’ training [72], and operating theatre teams [73], and in exploring ‘latent social threats’ in a labour and delivery unit [35]. This work illustrates both the exploration and improvement functions with the translational simulation framing. The examples draw upon broader theoretical and conceptual foundations than captured in the original translational simulation framing, including anthropology, relational coordination, and wider methodological approaches such as institutional ethnography [35] and participatory action research [44]. 

Our reflection on these published examples relates to the cultural signalling from our simulation design and delivery choices [41]. Perhaps the most important impact of integrating simulation into healthcare improvement strategies has been to send a powerful signal of commitment to constant improvement, based on developing a deep understanding of how work is done by frontline clinicians.

### Supporting healthcare improvement

There are conflicting conceptualisations in the healthcare improvement practitioner community as to whether simulation is a method [74], a technique, a research ‘test bed’ [75, 76], or an intersecting field of practice with healthcare improvement [77]. Conversations about these varied conceptualisations have been prompted by reports of simulation-based approaches to healthcare improvement, appearing in journals such as BMJ Quality and Safety [78, 79]. The methodologies described in these reports have been diverse, inconsistent, and variably cognisant of accepted quality improvement (QI) methodologies. These conversations appear (to us) to be surprisingly disconnected from parallel conversations in the healthcare simulation community [80]. 

The conceptual basis on which simulation is employed for quality improvement is evolving, reflecting developments in quality improvement practice. Examples such as improved time to intervention in acute stroke using a simulation-based intervention [57] are typical of a *linear* approach to quality improvement. In a linear approach, simulation is conceptualised as an intervention, with pre- and post-measures of performance used to measure intervention effectiveness, and researchers providing proof that simulation ‘works’ as an improvement tool. This conceptualisation, with its positivist paradigm, may be appropriate for some QI initiatives and for some research questions. The time to intervention in stroke markedly improved in the example given [57]. However, linear approaches often fail to reproduce successful outcomes when interventions are introduced into new contexts, as illustrated by the extensive literature pertaining to safety checklists in healthcare [81].

There are alternate conceptualisations by which healthcare simulation activities could be a cause, association, or even outcome of improvements in healthcare quality (82). This approach embraces an emerging ‘context logic’ in healthcare improvement practice and research: “*identifying the features of particular environments (such as organisational structures, processes, behaviours, practices, and values) that contribute to safety*” [82]. The notion of context logic is well-aligned with a core element of our conceptual stance, that translational simulation encompasses *exploring* work environments and the people in them and *shaping* the structural elements and social systems that affect performance in complex healthcare environments.

We suggest that the contribution of healthcare simulation to improving quality in healthcare goes well beyond simple technique and instead aligns with the conceptual framing offered in the 2017 translational simulation article [11], i.e. as a complex intervention [83]. This can encompass a plurality of potential conceptualisations for simulation - a method, a technique or a research test bed—appropriate to the context in which it is employed.

Our reflections on published examples of simulation for quality improvement are threefold. First, to fulfil our aspiration to healthcare simulation as an intersecting field of practice, our scholarship needs to shift from descriptions of project exemplars towards building consensus on theory and principles to guide practice [84]. Second, the evolution of our translational simulation *process* should align with contemporary and emerging approaches—such as the ‘context logic’ - in quality improvement practice. And third, our evolving conceptual model for translational simulation could better reflect the *conceptual intersection* with healthcare improvement, and practitioners could benefit from adopting the tools and *processes* from that field of practice.

## Terminology and conceptual overlaps

Other terminologies and conceptual framings have been offered in the academic conversations about what we have termed ‘translational simulation’. These terminologies include *In situ simulation, systems testing, systems integration, ‘sim QI’, and transformative simulation* [85]. In this section, we offer a brief description of some of these, with particular emphasis on how they may influence our evolving conceptual model.

### In situ simulation

In situ simulation (ISS) is delivered within the clinical environment [86]. It has been used to identify latent safety threats and improve health service outcomes where the team and system are closely linked [62, 63, 87]. Similar to the translational simulation literature, a review of published ISS examples reveals little consistency in approach: the contexts and aims are diverse [88]. Problematic in this descriptor is the preoccupation with ‘where’ (the location) of the simulation activity, rather than ‘why’ (its purpose) [11]. However, more recent literature has shifted the academic conversations toward the simulation *process* and underpinning *conceptual foundations*. Baxendale et al. generated a conceptual model for ISS in healthcare settings from a scoping review of ISS publications from 2008 to 2018 [89]. The review synthesised various principles, theories and approaches described in ISS literature. The proposed conceptual model consisted of four elements: Understand events, Design and Testing, Practice, and Assess/Evaluate [89]. Baxendale et al. aligned each element with key concepts, including complexity science, systems engineering, complex adaptive systems and knowledge transfer [89]. This model is the most comprehensive integration of *purpose*, *process* and *conceptual foundations* published to date, and informs our revised conceptual framing of translational simulation.

### Systems testing and ‘systems-focused simulations

Dube et al. define systems-focused simulations (SFS) by *process* and *purpose:* “both routine and high-risk situations are simulated, using real equipment, team members, environments, and processes” [32]. The aim is to “… facilitate the identification of safety threats, inefficiencies, and opportunities for quality improvement at all levels of the system and can aid in highlighting and reinforcing system resilience and organizational learning from simulation” [32]. This terminology has been used in reporting simulations to test new facilities [68], blood transfusion safety and policy [90], and post-cardiac surgery cardiac arrest protocols [91]. Dube’s article clearly articulates the challenges of implementing and integrating simulation within complex healthcare systems, and the need for robust methods of project management, stakeholder engagement, change management, and evaluation metrics [32, 92].

### Systems integration

*Systems integration* is defined in the Society for Simulation in Healthcare (SSH) healthcare simulation dictionary as “a category of simulation program accreditation that recognises programs that demonstrate consistent, planned, collaborative, integrated, and iterative application of simulation-based assessment, research, and teaching activities with systems engineering and risk management principles to achieve excellent bedside clinical care, enhanced patient safety, and improved outcome metrics across the health care system(s)” [93]. This descriptor encompasses both the *purpose* and *process* for simulation. The accreditation standards referred to are under the auspices of the US-based SSH [94]. They describe a variety of methods by which these aims may be achieved, drawing heavily on systems engineering principles and tools, including the Systems Engineering Initiative for Patient Safety (SEIPS) model of work system and patient safety [95, 96]. The accreditation standards mandate baseline consistency but do not necessarily reflect the practices of programs engaging in context-relevant approaches or innovations. Reports of simulation to test protocols and systems use this terminology of “systems integration” [91, 97], as do some descriptions of simulation debriefing methods adapted for this purpose [98].

### Transformative simulation

There have been attempts to align terminology for simulation that is focused directly on improving healthcare quality and safety. In reviewing the literature on ‘non-pedagogical’ simulation, Weldon and colleagues identified 68 different terms used, and coined the term ‘transformative simulation’—“to describe simulation as a tool to transform health and care through collective understanding, insight and learning, and to distinguish it from the more traditional educational/pedagogical approaches that are more commonly practised, or from specific system-focussed applications only” [85]. The authors developed a taxonomy of transformative simulation, categorising these activities by their objective. They described seven “simulation-based I’s”: identification, influence, improvement, involvement, inclusion, intervention, and innovation [85], but found it difficult to identify only one objective in many published examples. This calls into question the utility of such a granular taxonomy but underlines the need for *purpose* to remain central to any conceptual framing for healthcare simulation.

### The way forward for terminology?

Diverse terminologies and conceptual heterogeneity are no surprise in emerging and evolving fields of practice. This is particularly likely when healthcare simulation draws upon a plethora of theoretical and conceptual foundations. Common to translational simulation, in situ simulation, systems testing, systems integration, ‘sim QI’, transformative simulation and other terminologies is a direct focus on healthcare safety, quality, and systems as *purpose*. Also common is the struggle to determine how these purposes can be achieved; what is the optimal *process* for simulation design, delivery and implementation. In searching for these methods, many authors have unveiled *conceptual* models and connected them with fields of practice that can inform those methods: healthcare improvement, design thinking, systems science, change management, organisational behaviour and many others.

In our use of the term translational simulation, we mean a conceptual framing, rather than a technique, taxonomy or label. We embrace and encourage ongoing work toward consistency in terminology [47, 85, 89, 99], and view that as an important part of an evolving conceptual model.

## The developing translational simulation conceptual model: purpose, process and conceptual foundations

**Fig. 1:**
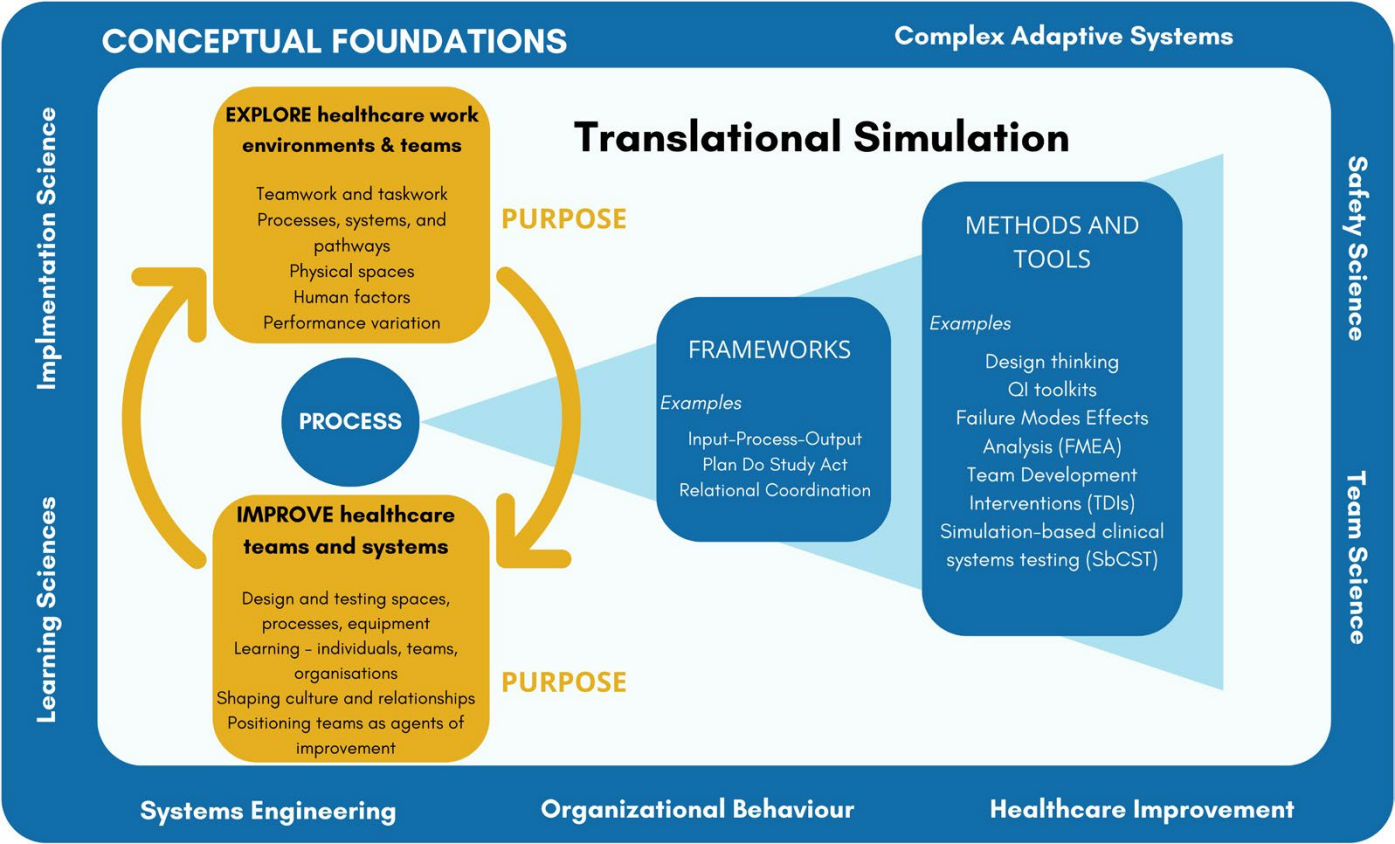
Translational simulation: purpose, process and conceptual foundations

The conceptual framing for how healthcare simulation contributes to improving healthcare quality and safety remains incomplete.*** “***Translational simulation: not where but why” [11] advocated a refocus on the *purpose* of simulation activities, against the tide of nomenclature relating to process, location and technique. Subsequent application in practice has underlined the strengths of that stance, while also highlighting limitations. Recent literature reviews have underpinned the need for clear framing, given the emergence and adoption of translational simulation [85]. These final sections of this article are forward-looking; here we propose an evolving conceptual model (Fig. 1) and describe how it supports simulation practitioners to effectively improve healthcare quality and safety.

*Purpose* remains central to translational simulation; exploring and improving healthcare environments, systems and teams. Translational simulation *process* is illustrated in two layers—broad *frameworks*, subsequently expanded to more specific *tools and methods*. This offers a level of practical detail to guide practitioners toward effective translational simulation design and implementation, while not being limited to a contextually bound ‘prescription’. Expanded theoretical and *conceptual foundations* on which the conceptual model draws are included safety science, system engineering, complex adaptive systems, team science, experiential learning and implementation science. Education—individual, team and organisational learning—is embraced as an important element of our comprehensive framing for simulation contributing to healthcare improvement.

Descriptions of the purpose, process and conceptual foundations in the model are not intended to be exhaustive or comprehensive. Rather they reflect examples drawn from published literature and from our personal experience working within this community of practice.

### Purpose

**Fig. 2:**
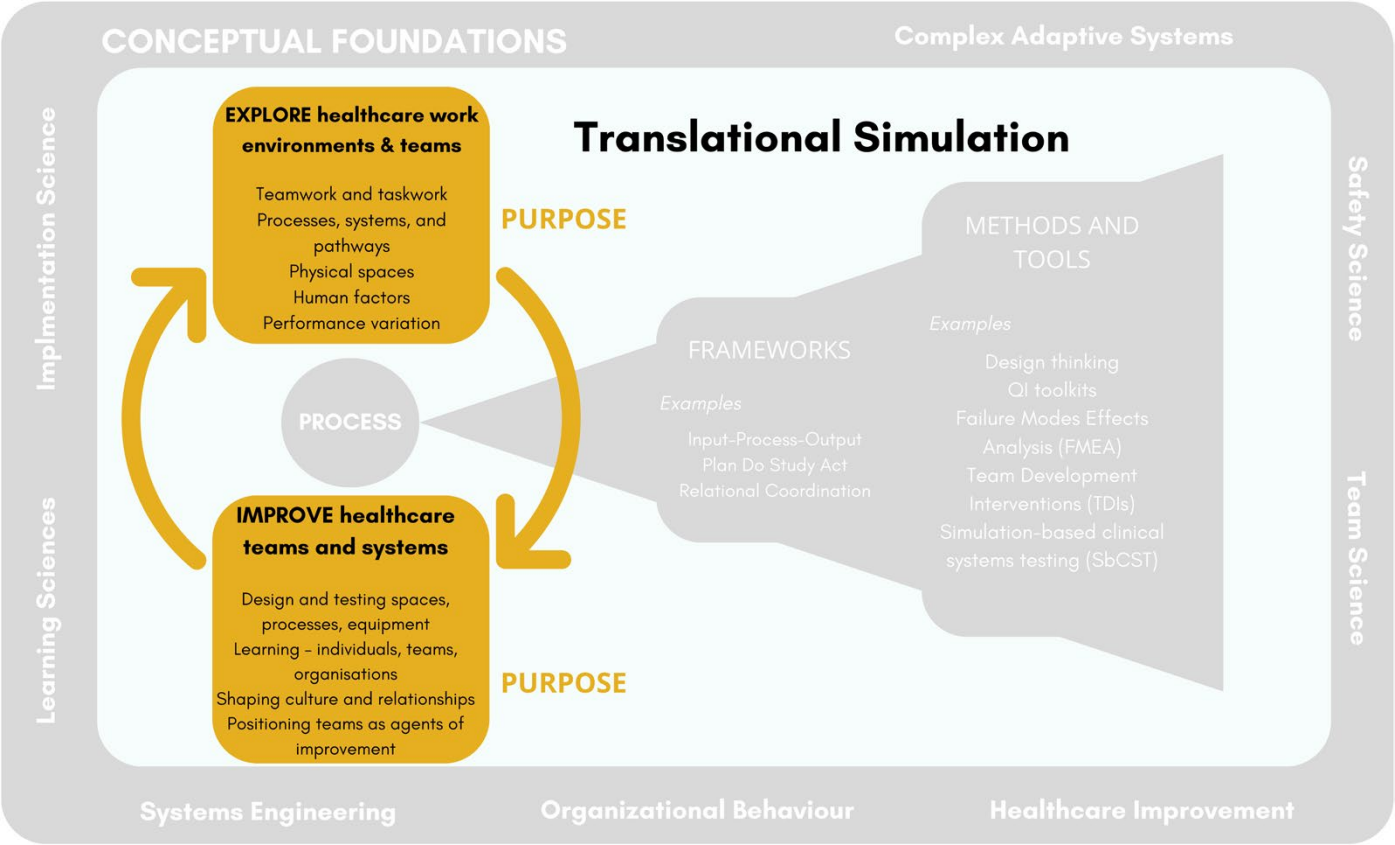
Translational simulation: purpose

The essence of translational simulation as a conceptual framing is *purpose -* improving healthcare quality, safety and systems (Fig. 2). Published examples of translational simulation (and those published under similar nomenclatures) strongly underpin this element of the model. We draw upon healthcare improvement in offering two distinct elements of purpose: exploration and improvement. The need to understand (explore) our complex adaptive healthcare systems is integral to improvement, but often neglected [82, 100, 101]. Our encouragement for healthcare simulation practitioners is to be mindful of the need for simulation to be employed to help explore and identify [47, 85, 89, 99] before leaping to fix, intervene or improve.

We propose that the shaping of healthcare team culture and relationships is an important mechanism of how simulation improves healthcare quality and safety. This has long been anecdotally experienced by simulation facilitators as a positive outcome of simulation training. Recent scholarly work has, however, embraced culture and relationships as a *purpose* for simulation, and sought to explore the methods and theoretical frameworks that might guide the *process* for achieving that aim [35, 40–42, 44, 46, 85]. This aligns with conversations in the healthcare improvement literature, namely, that healthcare professionals and teams are mediators of healthcare improvement, not just objects to be manipulated, or simply targets of that improvement [101].

We now see that one unintended consequence of promoting a conceptual framing of simulation toward a quality and safety purpose may have been the creation of a false dichotomy between ‘educational sim’ and ‘translational sim’. Terminology such as ‘non-pedagogical simulation’ [85] exacerbates the problem. In many ways, ironically, this unhelpful distinction, with its rush to label translational simulation as superior, seems to mirror the equally unhelpful debate about what is the superior place for simulation (i.e. in situ or in a sim lab). While such labelling may be necessary in some contexts (e.g. monthly reporting of activity for a simulation program), we argue that this runs counter to the more nuanced conceptual framing that is required, and which translational simulation calls for. Indeed, as a conceptual model, translational simulation holds that healthcare simulation focused on *educational* outcomes remains a dominant and necessary application within simulation practice, because the overarching outcome is quality, safety and systems: there is no dichotomy between the two. Education and learning contribute to quality and safety outcomes, albeit via the mechanisms of improving individual and team knowledge and skills, or by shaping attitudes and changing culture, and are situated within the *purpose* element of our conceptual model for translational simulation.

### Process

**Fig. 3:**
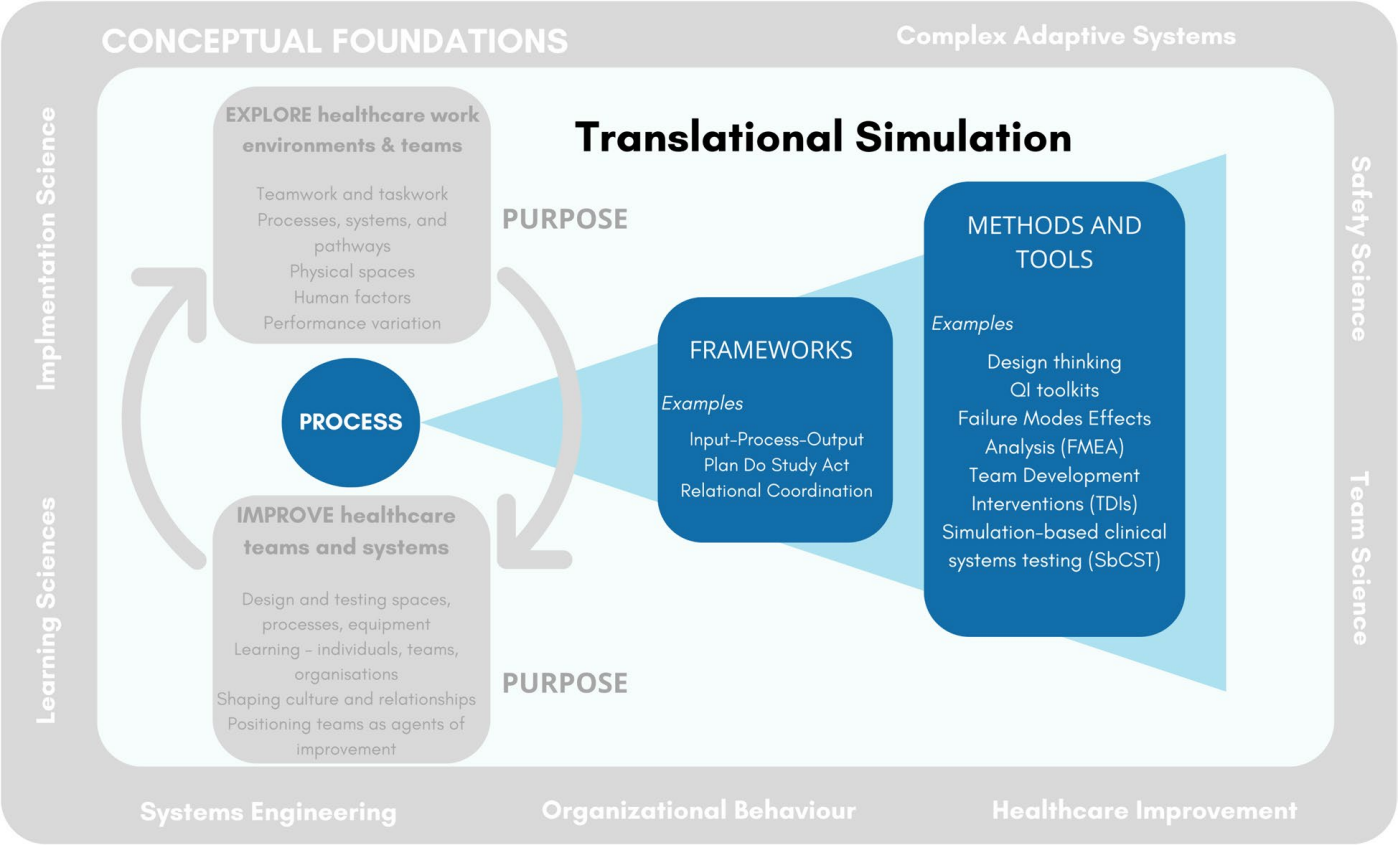
Translational simulation: process

While the term “translational simulation” offers a broad conceptual reframing of how simulation can contribute to healthcare improvement, the methods by which these goals are achieved are not well established or clearly elaborated. “Not where, but why?” may need to complement with “And how?” (Fig. 3). As demonstrated in our exploration of translational simulation in practice, published examples are mostly context-specific case studies [60–64], with simulation methods drawn from educational contexts or reliant on local resources and capacity. Efforts to distil broad principles and practical techniques have been published, but these tend to simply encourage the replication of approaches that may have led to success in one institution. Crystallising best practice is problematic when there is significant diversity in (1) the healthcare improvement targets encompassed by translational simulation, (2) the simulation techniques and professional expertise available, and (3) the contexts in which translational simulation may be applied. Indeed, ‘best practice’ is unlikely to be a worthy goal, given the dynamic and highly contextualised nature of the field.

Attempts have been made to “*describe a ‘road map’ for practitioners using translational simulation to address health service and patient-oriented outcomes*” [16]. Drawing on existing literature and personal experience, Nickson et al. offer an Input-Process-Output (IPO) framework for practitioners planning translational simulation activities, and hypothetical examples to illustrate how that framework could be applied in different contexts. They offer guiding principles for how translational simulation may be “conceptualised operationally”, including (1) Systems approach, embracing organisational learning principles, (2) Stakeholder engagement and participatory design, promoting engagement of frontline clinicians and healthcare consumers, (3) Strategy, not an event, emphasising that healthcare improvement requires an iterative and embedded approach, (4) Disciplined focus, recognising that “*goals are more likely to be achieved if they are narrow, specific, and well communicated to those designing and participating in the translational simulation activities*” [16], and (5) Functional task alignment, reflecting the diversity of simulation techniques and design choices, and how they should “*align with the objectives of the translational simulation strategy*” [16]. Other authors have proposed wide-ranging methodological approaches [32, 67–69, 71, 84, 92, 98, 102, 103], toolkits and ‘tips’ [104, 105] for simulation design, delivery and debriefing focused on quality and safety outcomes.

The process issues for translational simulation extend beyond simulation design and delivery. Integrating simulation meaningfully within health services requires attention to stakeholder engagement, change management and implementation (32, 48, 102], among many other factors. Guidance on how to position translational simulation programs (operationally) within healthcare institutions is emerging [15], but much more is to be learned. Safety has become a particular concern when simulation is conducted in clinical spaces, potentially leading to unintended threats to system integrity or patient safety [52, 106, 107]. Faculty development for translational simulation is embryonic, with even established programs taking predominantly informal approaches [108]. Strategies for program evaluation and demonstrating return on investment are diverse [84]. All of these issues, and many more besides, are important for translational simulation practice, and we leave them to others to develop as they further test and implement the translational simulation approach in their own contexts.

### Conceptual foundations and intersections

**Fig. 4:**
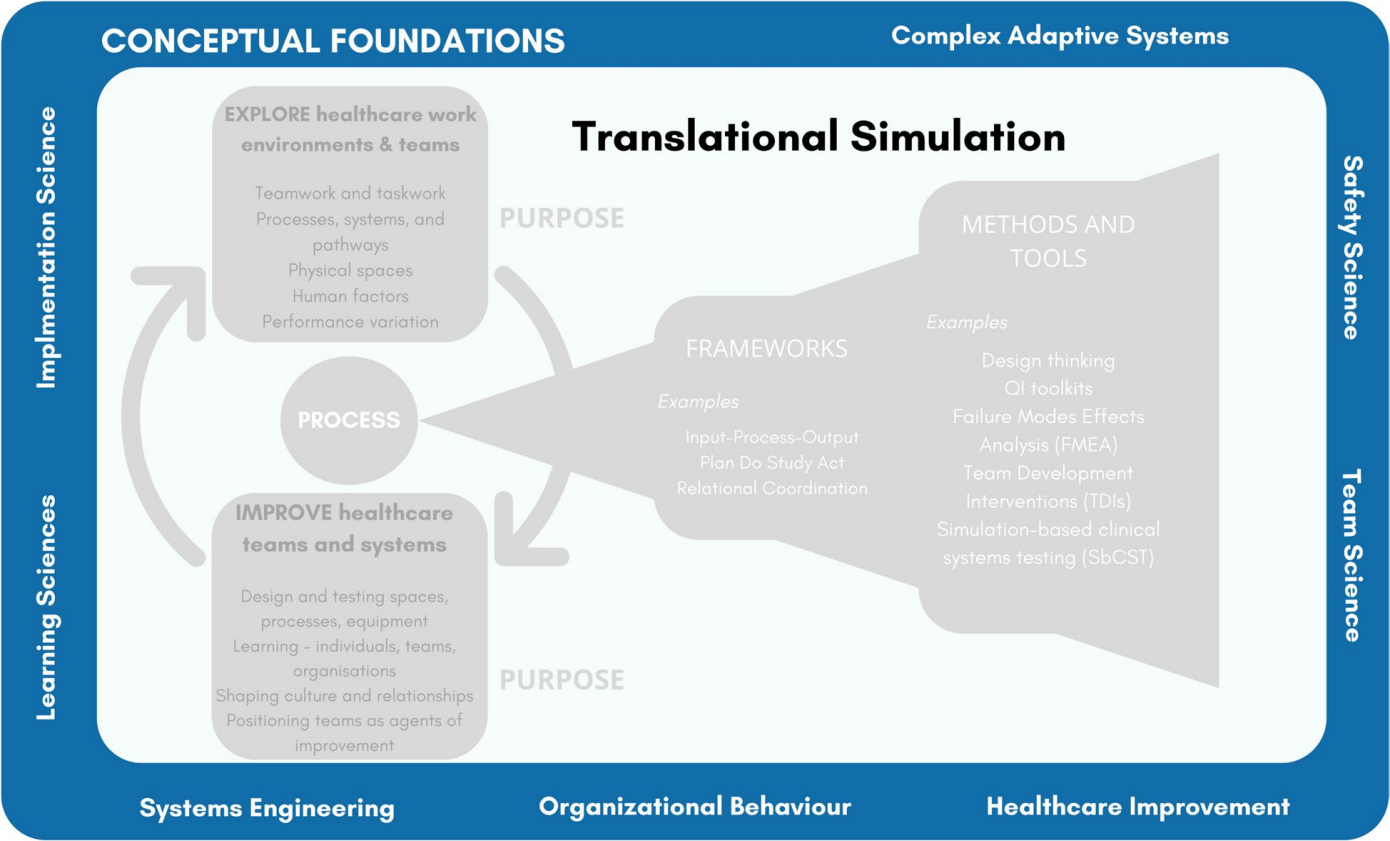
Translational simulation: conceptual foundations

Under-explored in the 2017 description of translational simulation were the conceptual and theoretical underpinnings of this emerging practice. The article [11] offered a model for what to do—diagnose, test, and improve processes and systems—as well as a justification for why to do it, but did not elaborate on intersecting fields of practice, including quality improvement, complex adaptive systems, systems modelling, experiential learning, and organisational learning. Subsequent publications have encouraged more thoughtful use of theoretical and conceptual models [32, 47, 75, 89]. This work is particularly important, as the bias toward action and technique in the healthcare simulation community is powerful. This bias has meant that existing and proven theoretical perspectives are not always thoughtfully integrated into research or practice [17]. Our illustration of some relevant conceptual foundations (Fig. 4) aims to encourage practitioners to reflect on these as they operationalise translational simulation concepts, and to continue to consider other theoretical foundations which may be appropriate.

Exploration of broader theoretical and conceptual foundations by simulation practitioners has led to the intersection with a wider range of communities of practice. Systems engineers [32, 95], architects [71], design experts [69], healthcare improvement specialists [75, 84, 100], safety science experts [103], institutional ethnographers [35, 82], and many others are key collaborators for translational simulation research, practice and faculty development [108]. These communities of practice help connect to wider conceptual and theoretical considerations, consistent with the traditions of the broader healthcare simulation field [49]. As translational simulation continues to spread, undoubtedly so will these connections—which we encourage.

## Conclusion

Translational simulation remains an incomplete framing for how simulation contributes to healthcare quality and safety. We have described evolving practice in this area over the last 6 years, illustrating diverse applications and methodologies. We have crystallised this in a graphical representation of this conceptual framing with three core elements: purpose, process and conceptual foundations.

We hope that this clarity supports the work of simulation practitioners and scholars, and informs colleagues from intersecting fields of practice. Now, as simulation continues to develop and mature as a field of its own, and as simulation becomes more embedded in the complex systems of healthcare in the myriad of cultural, economic, geographic, and regulatory frameworks, we hope this more elaborated conceptual framing will support colleagues seeking to improve the quality and safety of patient care through simulation. As we look back at what has been done since 2017, and as we seek to frame our own work over the coming years, we found it both necessary and helpful. We look forward to working on translational simulation implementation, faculty development, ethical issues, evaluation and many other important issues for optimising the contribution to healthcare quality and safety.

## Data Availability

Not applicable.

## Abbreviations

ISS: In situ simulation
FMEA: Failure Modes Effects Analysis
SbCST: Simulation-based clinical systems testing
QI: Quality improvement
SFS: Systems focused simulations
SSH: Society for Simulation in Healthcare
SEIPS: Systems Engineering Initiative for Patient Safety
IPO: Input-process-output
